# Effectiveness of an Alcohol Screening and Brief Intervention for Low-Income Clients Drinking at a High-Risk Level in Social Service Settings

**DOI:** 10.1192/j.eurpsy.2022.2153

**Published:** 2022-09-01

**Authors:** Y. Kim, S.K. Chung, E.S. Choi, S.Y. Park

**Affiliations:** 1 The Catholic University of Korea, Department Of Social Welfare, Graduate School Of Addiction Studies, Bucheon-si , Korea, Republic of; 2 Chung Ang University, Faculty Of Social Welfare, Seoul, Korea, Republic of; 3 The Catholic University of Korea, Graduate School Of Addiction Studies, Gwacheon-si , Korea, Republic of; 4 The Catholic University, Graduate School Of Addiction Studies, Seoul, Korea, Republic of

**Keywords:** alcohol screening and brief intervention, ASBI, low-income clients, high-risk drinking

## Abstract

**Introduction:**

Alcohol screening and brief interventions (ASBIs) for risky drinkers are known to reduce alcohol consumption and alcohol-related harm. The present study was the first to investigate the effectiveness of an ASBI for high-risk drinkers of low socioeconomic status (SES) in the Korean community social service setting.

**Objectives:**

This study aims to evaluate the effectiveness of an ASBI for clients in community social service settings in South Korea.

**Methods:**

A total of 153 clients in social service agencies participated in this study. Clients in the experimental group received alcohol use screening and two sessions of brief motivational interventions (MI). Clients in the comparison group received alcohol problems screening test only. Primary outcome variable was the amount of weekly alcohol consumption, which was measured once before the intervention and three times after the intervention.

**Results:**

When analyses were conducted separately for participants from the self-sufficiency centers and those from the community welfare centers, there was a significant time and group interaction effects. The amount of weekly alcohol consumption of the experimental group was gradually reduced over time. However, the amount of the comparison group was reduced at the four-week follow-up but was increased both at the eight-week and 12-week follow-ups.

**Conclusions:**

This study demonstrates the need to provide training and education in the ASBI to social service workers working with the underprivileged, as such training would increase the identification of alcohol-related risks of the people most vulnerable to alcohol-related problems.

**Disclosure:**

No significant relationships.

